# Sodium-glucose cotransporter 2 inhibitors and mitochondrial functions: state of the art

**DOI:** 10.17179/excli2022-5482

**Published:** 2023-01-04

**Authors:** Habib Yaribeygi, Mina Maleki, Alexandra E. Butler, Tannaz Jamialahmadi, Amirhossein Sahebkar

**Affiliations:** 1 Research Center of Physiology, Semnan University of Medical Sciences, Semnan, Iran; 2 Urology and Nephrology Research Center, Shahid Beheshti University of Medical Sciences, Tehran, Iran; 3 Research Department, Royal College of Surgeons in Ireland Bahrain, Adliya, 15503, Bahrain; 4 Applied Biomedical Research Center, Mashhad University of Medical Sciences, Mashhad, Iran; 5 Surgical Oncology Research Center, Mashhad University of Medical Sciences, Mashhad, Iran; 6 Biotechnology Research Center, Pharmaceutical Technology Institute, Mashhad University of Medical Sciences, Mashhad, Iran; 7 School of Medicine, The University of Western Australia, Perth, Australia; 8 Department of Biotechnology, School of Pharmacy, Mashhad University of Medical Sciences, Mashhad, Iran

**Keywords:** sodium-glucose cotransporter 2 inhibitors, mitochondria, diabetes mellitus, mitophagy, oxidative stress

## Abstract

Sodium-glucose cotransporter 2 inhibitors (SGLT2is) are a class of newly introduced antidiabetic drugs with potent hypoglycemic effects. Recent evidence suggests that these drugs have extraglycemic impacts and are therefore able to provide additional benefits beyond glucose lowering. Mitochondrial dysfunction is a central facet of many disorders that negatively impacts many tissues and organs, especially in the setting of diabetes. Therefore, it would be hugely beneficial if an antidiabetic drug could also provide mitochondrial benefits to improve cellular function and reduce the risk of diabetic complications. In this review, we have surveyed the literature for possible mitochondrial benefits of SGLT2is and we discuss the possible mechanisms involved.

## Introduction

The global prevalence of diabetes mellitus (DM) is rising rapidly (Divers et al., 2020[16]). DM is the most prevalent metabolic disorder worldwide and negatively impacts major physiological systems leading to disrupted homeostasis (Eid et al., 2019[18]; Divers et al., 2020[16]; Imai, 2021[35]). Uncontrolled DM underlies many cardiovascular, neuronal, retinal, renal and dental conditions especially those classified as “diabetic complications” (Forbes and Cooper, 2013[26]). Although the exact pathophysiology of DM-induced disorders has not been fully elucidated (Forbes and Cooper, 2013[26]), the role of mitochondrial dysfunction is very important (Sharma, 2015[67]; Hallan and Sharma, 2016[31]) and known to be involved in chronic kidney disease (Hallan and Sharma, 2016[31]), cardiovascular complications (Chistiakov et al., 2018[9]), neuropathies (Fernyhough, 2015[23]) and retinal diseases (Barot et al., 2011[3]). Mitochondrial dysfunction can induce and/or promote oxidative injuries, a major pathological insult, as well as inflammatory and apoptotic processes (Huang et al., 2019[34]; Picca et al., 2020[58]). Therefore, normalizing mitochondrial function may help to restore tissue homeostasis and prevent diabetic complications (Parmar et al., 2022[56]). 

Sodium-glucose cotransporter 2 inhibitors (SGLT2is) are a class of newly introduced antidiabetes medications that provide potent glucose-lowering outcomes (Davidson and Kuritzky, 2014[13]; Yaribeygi et al., 2019[80]). They act by inducing urinary glucose excretion by the kidneys (Yaribeygi et al., 2019[82]). However, recent evidence suggests that these drugs have pleiotropic actions (Yaribeygi et al., 2020[79], 2022[83][84]; Masson et al., 2021[50]) and are able to modulate mitochondrial function and inhibit or suppress pathophysiologic pathways induced by mitochondrial dysfunction in the setting of diabetes (Maejima, 2020[48]; Mone et al., 2022[52]). However, the exact pathways involved are not fully understood. In this current study, we discuss the evidence supporting SGLT2 inhibitor effects on mitochondrial function and explain the possible mechanisms involved.

## Sodium-Glucose Cotransporter 2 Inhibitors

**Figure 1:**
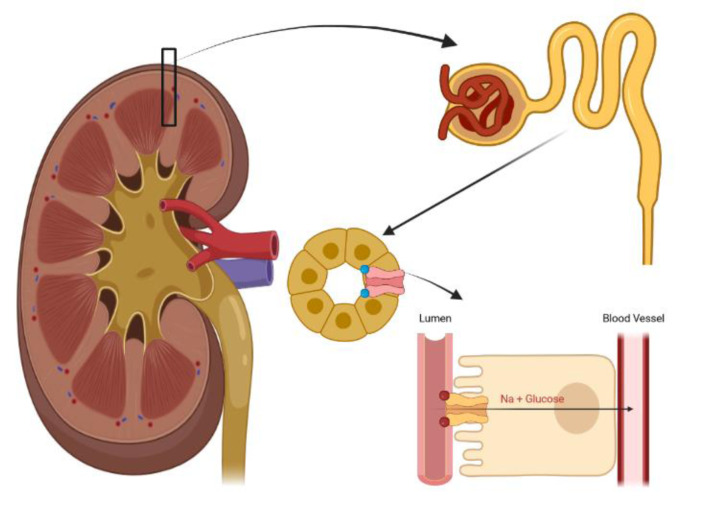
The mechanism of SGLT2 activity in the renal proximal tubule. SGLT2 inhibitors inhibit SGLT2 activity and induce urinary glucose and sodium excretion.

SGLT2 inhibitors are a class of newly-introduced glucose lowering drugs that reduce serum glucose by inhibition of tubular glucose reabsorption and induction of urinary glucose excretion (Davidson and Kuritzky, 2014[13]; Yaribeygi et al., 2019[80]). Sodium-glucose cotransporters exist as two forms of active cotransporters, type 1 and type 2, are mainly located in S2 and S3 segments of renal proximal tubules (as well as in the intestine) and reabsorb the majority of filtrated urinary glucose (Yaribeygi et al. 2019[80][82]) (Figure 1(Fig. 1)). SGLT2is inhibit this process and induce glycosuria completely independent of the insulin hormone (Chao, 2014[5]). Since discovery of the first SGLT2 inhibitor, phlorizin, several forms of these drugs have been introduced which all reduce the blood glucose near to the level of the capacity of nephrons for glucose reabsorption (Chao and Henry, 2010[6]; Clar et al., 2012[10]). Beyond their potent glucose-lowering effects, they have other pharmacological effects such as glycogenesis suppression, neuroprotection, reduction of epicardial adiposity, improvement of peripheral tissue insulin sensitivity, enhancement of the glucagon release response and induction of insulin secretion from pancreatic islet beta cells (Han et al., 2008[32]; Ferrannini et al., 2014[24]; Wilding et al., 2014[76]; Kern et al., 2016[36]). Canagliflozin, dapagliflozin and empagliflozin are well known forms of SGLT2 inhibitors (Reddy and Inzucchi, 2016[60]). Use of these drugs may, however, be accompanied by some adverse effects, such as dehydration, dizziness, hypotension, urinary tract infections and fainting (Reddy and Inzucchi, 2016[60]).

## Physiology of Mitochondria

Mitochondria are double-membraned cellular organelles that are closely involved in important cellular processes including energy production (as ATP [adenosine triphosphate]), calcium storage, fatty acid oxidation and production, heat production, cell survival and apoptosis, and cell signaling (Balaban et al., 2005[1]; Elrod and Gustafsson, 2018[20]). These highly conserved organelles have two layers of membrane (outer and inner) and two spaces (the intermembrane space that lies between the two membranes and the matrix which is enclosed by the inner membrane) (van Vliet et al., 2014[71]). Mitochondria produce energy through a series of sequential steps that occur in the mitochondrial respiratory chain (MRC), also known as the mitochondrial electron transport chain (METC), through a process termed "oxidative phosphorylation" (DiMauro and Schon, 2003[15]). Oxidative phosphorylation is an oxygen-dependent biochemical process triggered by pyruvate entering into the mitochondria and ATP and water production in the tricarboxylic acid (TCA) (Krebs's) cycle which takes place in the matrix of the mitochondrion (García‐Ruiz and Fernández‐Checa, 2018[28]). This process involves passing electrons from donors (which are at lower redox potential) to acceptors (which are at higher ones) (DiMauro and Schon, 2003[15]). Located and embedded in a tightly folded membrane of the inner mitochondrial membrane, the MRC is composed of five separate complexes, complexes I-V, all of which have their own subunits, as well as two small carriers of electrons, ubiquinone (or coenzyme-Q10) and cytochrome-C (DiMauro and Schon, 2003[15]; van Vliet et al., 2014[71]). These complexes together create an electrical charge differential between the two sides of the mitochondrial inner membrane, a factor critical in ATP production since this provides the required driving force for proton transfer across the inner membrane (DiMauro and Schon, 2003[15]). In brief, ATP synthesis is achieved through two separate processes: first, electron transfer along complexes to molecular oxygen and generating a water molecule and, second, pumping the protons at the same time across the inner membrane of mitochondria by complexes I, III, and IV (DiMauro and Schon, 2003[15]). The generated ATP results from the influx of these protons back into the matrix space (surrounded by inner membrane) via complex V (also known as ATP synthase complex) (DiMauro and Schon, 2003[15]; Vyas et al., 2016[74]; Elrod and Gustafsson, 2018[20]). 

Mitochondria possesses its own genetic material, mitochondrial DNA (mtDNA), that is a double-stranded circular DNA of 16569-bp (base pairs) encoding 37 genes as well as its own RNA synthesizing machinery (Taanman, 1999[68]; Elrod and Gustafsson, 2018[20]). However, mitochondria additionally require some proteins encoded by the nucleic genome that enter from the cytosol into the mitochondria (Elrod and Gustafsson, 2018[20]). Therefore, mitochondrial proteins are under both Mendelian and genetic control and, thus, delineating the exact underlying genetic cause of mitochondrial disorders is complex (DiMauro and Schon, 2003[15]).

## Role of Mitochondria in Health and Diseases

Since mitochondria produce the majority of energy needed for cell survival (about 13 times more that glycolysis), these organelles are known as the "powerhouses" of the cells and have critical importance (Elrod and Gustafsson, 2018[20]). They are found in most eukaryotic cells but are concentrated in cells of highly-oxidative tissues such as kidneys and liver (Hall, 2015[30]; Elrod and Gustafsson, 2018[20]). The mitochondria produce ATP via the oxidative-phosphorylation process in a two-step process (1) oxidation of electron donors (NADH or FADH_2_) providing the necessary electrons for mitochondrial electron transport chain (METC) and (2) phosphorylation of ADP to ATP (Fakhruddin et al., 2017[21]). Mitochondrial dysfunction is a state of reduced efficiency of MRC and lower levels of ATP synthesis (Barcelos et al., 2019[2]). It is commonly due to an inadequate number of mitochondria, inadequate metabolic substrates, or a defect in the MRC and ATP-synthesis machinery (Nicolson, 2014[53]). 

Mitochondrial dysfunction and reduced ATP synthesis is closely related to the pathophysiology of neurodegenerative diseases, such as Alzheimer's disease (AD), Parkinson's disease (PD), Huntington's disease (HD) and amyotrophic lateral sclerosis (ALS), DM and metabolic syndrome, cardiovascular complications, such as atherosclerosis and chronic heart disease (CHD), autoimmune disorders, such as multiple sclerosis (MS), Systemic Lupus Erythematosus (SLE), rheumatoid arthritis (RA) and type 1 DM, hepatic complications such as fibrosis and cirrhosis, and neurobehavioral and psychological disorders (Nicolson, 2014[53]; Chen et al., 2018[7]; Schuster et al., 2018[65]; Barcelos et al., 2019[2]; Perez Ortiz and Swerdlow, 2019[57]; Pinti et al., 2019[59]; Xu et al., 2020[78]; Manolis et al., 2021[49]). In addition, mitochondrial dysfunction is related to excess fatigue and reduced respiratory capacity (Nicolson, 2014[53]). Considering the vital roles of these organelles, their integrity and normal function are critical to maintaining body homeostasis. 

## Effects of SGLT2 Inhibitors on Mitochondrial Function

In the setting of diabetes, there are commonly different degrees of mitochondrial dysfunction (Pinti et al., 2019[59]). These deficits are intimately involved in the pathophysiology of diabetes-induced complications (Pinti et al., 2019[59]). Further, it has suggested that improvement in mitochondrial function may lead to improvement of cellular activities and prevent or mitigate diabetes complications (Teodoro et al., 2019[70]; Kusminski et al., 2020[39]). Therefore, we will review current knowledge about the possible beneficial or unfavorable effects of SGLT2 inhibitors on mitochondrial function (Table 1(Tab. 1); References in Table 1: Croteau et al., 2021[11]; Durak et al., 2018[17]; Hawley et al., 2016[33]; Lee et al., 2019[40]; Li et al., 2019[41], 2022[42]; Maejima, 2019[47]; Mizuno et al., 2018[51]; Mone et al., 2022[52]; Santos-Gallego et al., 2019[63]; Takagi et al., 2018[69]; Verma et al., 2018[72]; Zhou et al., 2018[86]).

### Mitochondrial Reactive Oxygen Species production 

Mitochondria are a major source of reactive oxygen species (ROS) as well as nitrosative oxygen species (NOS) (García‐Ruiz and Fernández‐Checa, 2018[28]; Elfawy and Das, 2019[19]). These highly reactive free radicals, that are produced physiologically as by-products of oxidative phosphorylation, can damage cellular elements and induce and promote apoptosis, fibrosis and other pathological cellular events (Elfawy and Das, 2019[19]). Since they are potent inducers of cellular damage, maintaining mitochondrial ROS production within physiologic limits is very important (García‐Ruiz and Fernández‐Checa, 2018[28]). Major causes of mitochondrial ROS production are (1) increased electron delivery to METC due to more electron donors, (2) leaking electrons, (3) suppression of mitochondrial antioxidant elements, (4) a mutation in the mtDNA (Nishikawa et al., 2000[54]; Sakai et al., 2003[61]). In the diabetic milieu, the amounts of nicotinamide adenine dinucleotide (NADH) and flavin adenine dinucleotide (FADH_2_) are increased through glycolysis and the Krebs cycle (Giacco and Brownlee, 2010[29]; Fakhruddin et al., 2017[21]). This leads to an excess of electron donors and increased electron delivery to complex I with more anion superoxide generation (Liu et al., 2002[43]; Yaribeygi et al., 2019[82]). However, other mechanisms are also involved in mitochondrial oxidative stress in the setting of diabetes (Yaribeygi et al., 2019[81]).

**Figure 2:**
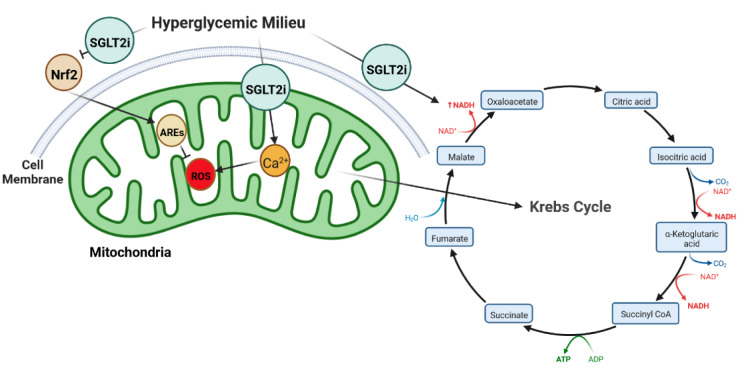
SGLT2 inhibitors reduce mitochondrial ROS production by at least 3 pathways as 1. Damping the inhibitory effects of hyperglycemia on Nrf2 signaling, 2. Reduction in NADH level produced by Krebs cycle (as substrate of ROS generation), and 3. Reduction in mitochondrial Ca^2+^ overload (which initiates ROS production).

Available evidence suggests that SGLT2 inhibitors ameliorate mitochondrial ROS production (Figure 2(Fig. 2)) (Mone et al., 2022[52]). Mone et al. recently found that SGLT2 inhibition improves endothelial cell function (Mone et al., 2022[52]). They showed that 3 months of empagliflozin therapy reduces mitochondrial Ca^2+^ overload and ROS production, leading to improved vascular function in diabetic patients (Mone et al., 2022[52]). Li and coworkers reported that SGLT2 inhibitor therapy resulted in a reduction in myocardial oxidative stress injury and cardiac fibrosis in diabetic mice (Li et al., 2019[41]). They found that empagliflozin therapy for 8 weeks improves myocardial structure and function by controlling myocardial oxidative stress through inhibition of the TGF-β (transforming growth factor β)/Smad pathway and activation of Nrf2/ARE (nuclear erythroid 2-related factor 2/antioxidant response element) signaling pathways (Li et al., 2019[41]). Moreover, Zhou and coworkers showed that empagliflozin inhibits ROS production and mitochondrial oxidative stress (Zhou et al., 2018[86]). They demonstrated that 20 weeks of empagliflozin therapy preserved cardiac microvascular barrier function and integrity and prevented DM-induced microvascular complications in the myocardium of diabetic animals by improving cardiac endothelial cell function by suppression of mitochondrial oxidative stress in treated mice (Zhou et al., 2018[86]). A more recent study has provided further evidence suggesting that empagliflozin is able to improve cardiac function by reducing ROS production and mitochondrial oxidative damage in a non-diabetic model of pressure overload-induced-heart failure mice (Li et al., 2022[42]). The glucose-lowering effects of these drugs is another possible link between SGLT2 inhibition and mitochondrial ROS, since lowering the prevailing glucose level reduces the amount of produced electron donors such as NADH and FADH_2_ (Llorens-Cebrià et al., 2022[44]). Collectively, these reports strongly suggest that SGLT2 inhibitor therapy can restore METC activity and reduce mitochondrial oxidative stress (Yaribeygi et al., 2019[80]). Therefore, SGLT2 inhibitors can modulate and normalize mitochondrial function through a number of different pathways (Figure 2(Fig. 2)).

### Mitochondrial count, mitochondrialbiogenesis and mitophagy 

The number of active mitochondria is directly related to the rate of oxidative phosphorylation and ATP synthesis and, therefore, tissue performance and activity (Witte et al., 2009[77]). As such, many complications, including organ failure, result from an elevated rate of mitophagy resulting in a reduced mitochondrial count (Witte et al., 2009[77]; Chinta et al., 2010[8]). Most diabetic complications, examples being diabetic nephropathy and atherosclerosis, are associated with a reduced mitochondrial mass and lowered rate of ATP synthesis (Kitada et al., 2011[37]; DeBarmore et al., 2020[14]). Therefore, improvement in mitochondrial survival and the normalization of mitochondrial mitophagy could prevent or delay the diabetic complications and organ failure (Yoon et al., 2011[85]).

SGLT2 inhibitors have been shown to improve mitochondrial number and mitophagy (Maejima, 2019[47]). Maejima reported that SGLT2 inhibitors play a protective role in cardiomyocytes, at least in part, by promoting mitophagy (Maejima, 2019[47]). He has demonstrated that empagliflozin improves cardiomyocyte activity through binding with non-SGLT2 protein(s) localized in mitochondria and normalizing mitophagy in cultured cardiomyocytes (Maejima, 2019[47]). Mizuno et al. reported that empagliflozin provided cardioprotective effects by suppressing the hyperglycemia-induced decrease in mitochondrial size and number through improving the intracellular antioxidant defense system (Mizuno et al., 2018[51]). They found that empagliflozin therapy normalized the number and size of mitochondria and prevented a reduction in mitochondrial size after myocardial infarction through inhibition of oxidative stress and restoration of autophagy in heart tissue from diabetic rats (Mizuno et al., 2018[51]). Further, Vettor et al. suggested that empagliflozin provided some cardio-protective effects by inducing mitochondrial biogenesis via a model of energy wasting through glycosuria (Vettor et al., 2017[73]). Li and colleagues recently reported that empagliflozin induced mitochondrial biogenesis and restored normal mitochondria morphology in non-diabetic mice (Li et al., 2022[42]). Also, they observed that SGLT2 inhibition increased the mitochondrial count, prevented apoptosis and induced autophagy in cultured cardiac fibers of mice (Li et al., 2022[42]). Recent evidence indicates that SGLT2 inhibition with empagliflozin induced mitochondrial biogenesis in the hyperglycemic state (Lee et al., 2019[40]).

Takagi and coworkers demonstrated that ipragliflozin reversed high-fat diet-induced morphological damage in renal tubular cells of mice (Takagi et al., 2018[69]). They found that ipragliflozin causes this effect via induction of OAF-1 (optic atrophy protein 1) and MFN-1 (Mitofusin 1) expression (Takagi et al., 2018[69]). Empagliflozin has been shown to reduce mitochondrial fragmentation and induce autophagy by normalizing the ATP/ADP ratio in cultured hRPTCs (human renal proximal tubular cells) (Lee et al., 2019[40]). Durak and coworkers demonstrated that dapagliflozin alters expression levels of mitochondrial fusion/fission protein such as Mfn-1 (Mitofusin-1), Mfn-2, and Fis-1 (Mitochondrial fission 1 protein) (Durak et al., 2018[17]). Moreover, it has been suggested that SGLT2 inhibitors improve mitochondrial biogenesis by induction of important transcription factors, PGC-1 [peroxisome proliferator-activated receptor gamma co-activator 1-alpha] and TFAM [mitochondrial transcription factor A], thus regulating the balance between mitochondrial fusion and fission (Dabravolski et al., 2022[12]). SGLT2 inhibitors can also improve mitophagy through regulation of DNM1L [dynamin 1 like], FIS1 [fission, mitochondrial 1], MFN1, MFN2, and OAF-1, and improve mitochondrial integrity (Dabravolski et al., 2022[12]). Taken together, this evidence indicates that SGLT2 inhibitors are able to modulate mitochondrial biogenesis and improve their integrity and mass, leading to improvement in cellular respiration. 

### Metabolic efficiency, metabolic events and ATP production

As noted earlier, intact metabolic function of mitochondria is critical for cell survival and ATP production (Lowell and Shulman, 2005[45]). Altered mitochondrial metabolism and bioenergetics results in reduced mitochondrial respiration leading to enhanced mitochondrial ROS generation and increased mtDNA injury that, in turn, induce abnormal mitochondrial morphology (Dabravolski et al., 2022[12]). Reduced mitochondrial ATP production is a main feature of many diabetic complications (Nicolson, 2014[53]; Luptak et al., 2018[46]; Barcelos et al., 2019[2]). Decreased ATP production limits the ability of tissues to optimally function and impairs ATP-dependent enzymes involved in physiological activities (Croteau et al., 2021[11]). In the hyperglycemic state, pathological pathways, such as the hexosamine and polyol pathways, are induced, and mitochondria are at higher risk of metabolic dysfunction and a failure of their ATP synthesis machinery (Lowell and Shulman, 2005[45]; Nicolson, 2014[53]; Wada and Nakatsuka, 2016[75]; Forbes and Thorburn, 2018[27]). Therefore, improvement in these processes may help to reduce the diabetic complications that are dependent upon the level of ATP production (Lowell and Shulman, 2005[45]; Nicolson, 2014[53]; Wada and Nakatsuka, 2016[75]; Forbes and Thorburn, 2018[27]).

Some evidence has demonstrated that SGLT2 inhibitors enhance mitochondrial efficiency and improve bioenergetics status and ATP production (Sawicki et al., 2021[64]). For example, Verma and coworkers found that empagliflozin increased overall ATP production by ∼30 % in cardiac tissue of diabetic mice (Verma et al., 2018[72]). They revealed that empagliflozin exerts this effect by an increase in the rate of mitochondrial glucose and fatty acid oxidation in the treated animals (Verma et al., 2018[72]). Croteau et al. recently reported that empagliflozin therapy upregulated genes involved in fatty acid metabolism and mitochondrial oxidative phosphorylation and improved phosphocreatine/ATP ratio and ATP content in the myocardium of both diabetic and non-diabetic mice (Croteau et al., 2021[11]). Further recent evidence has demonstrated that empagliflozin improved bioenergetics status of myocardium tissues in non-diabetic pigs (Santos-Gallego et al., 2019[63]). In this study, empagliflozin therapy increases mitochondrial ATP content by switching myocardial fuel utilization away from glucose toward more energetic substrates as fatty acids, amino acids and ketone bodies (Santos-Gallego et al., 2019[63]). 

**Figure 3:**
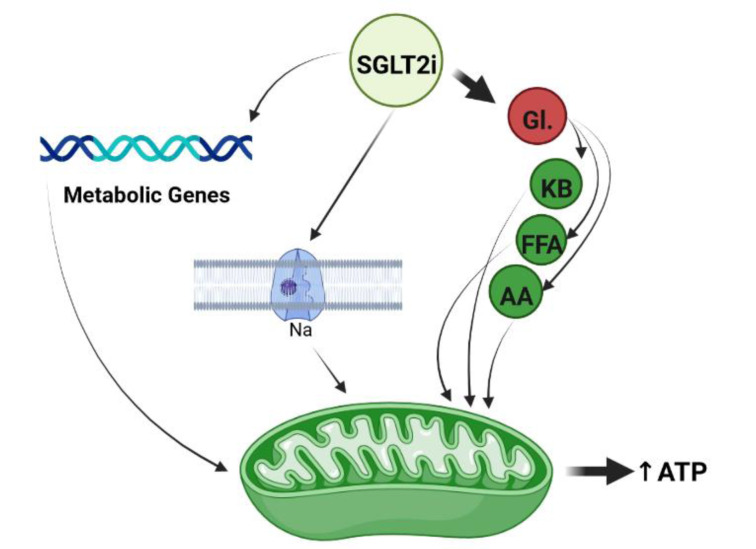
SGLT2 inhibition improves mitochondrial metabolism via 1. Switching fuel utilization from Gl. (glucose) to KB (ketone body), FFA (free fatty acid) and AA (amino acid), 2. Upregulation of metabolic genes, and 3. Increase in voltage-gated sodium currents in mitochondria

Hawley and coworkers found that canagliflozin (but not empagliflozin or dapagliflozin) improved metabolic processes and lipid oxidation in cultured human embryonic kidney (HEK)-293 cells by inhibition of complex I of the MRC, leading to increases in cellular AMP and ADP levels (Hawley et al., 2016[33]). Sa-Nguanmoo et al. demonstrated that dapagliflozin exerted neuroprotective effects and improved cognitive ability in high fat diet induced obese rats via potentiating mitochondrial efficiency and improving brain function (Sa-Nguanmoo et al., 2017[62]). Dapagliflozin was also able to positively modulate mitochondrial ion homeostasis and augment mitochondrial efficiency (Durak et al., 2018[17]). Durak and coworkers demonstrated that dapagliflozin improved mitochondrial efficiency by preserving MMP (mitochondrial membrane potential) and cytosolic Ca2+-homeostasis, with an increase in voltage-gated sodium currents and ADP/ATP ratio in cardiomyocytes (Durak et al., 2018[17]). Collectively, the evidence suggests that SGLT2 inhibitors are able to positively modulate bioenergetics status and metabolic pathways toward increased ATP production in mitochondria through several pathways (Figure 3(Fig. 3)). 

### Other possible pathways and adverse reports 

SGLT2 inhibitors may modulate mitochondrial functions through other potential mechanisms such as altering the mitochondrial membrane potential (Durak et al., 2018[17]; Komatsu et al., 2020[38]), and impacting cellular calcium and sodium handling (Bertero et al., 2018[4]; Filippatos et al., 2019[25]; Olgar et al., 2020[55]). However, there are very few adverse reports published in the literature. Secker and coworkers in 2018 found that canagliflozin inhibited complex I of METC and induced intracellular cytotoxicity by causing accumulation of glutamate, glutamine and alanine, so impairing mitochondrial function (Secker et al., 2018[66]). They issued a warning regarding the use of two types of SGLT2is, canagliflozin and dapagliflozin, in diabetic patients since these drugs can exert some nephrotoxic effects, as confirmed recently by the Federal Drug Administration (FDA) in the United States (FDA, 2017[22]; Secker et al., 2018[66]). Canagliflozin was shown to inhibit not only complex I of MRC, but also mitochondrial GDH (glutamate dehydrogenase) in cultured human renal tubular cells which may be relevant to some of the observed nephrotoxic side-effects (Secker et al., 2018[66]). Consequently, more investigation is required to fully delineate the potential mitochondrial benefits of SGLT2 inhibitors.

## Conclusion

**Figure 4:**
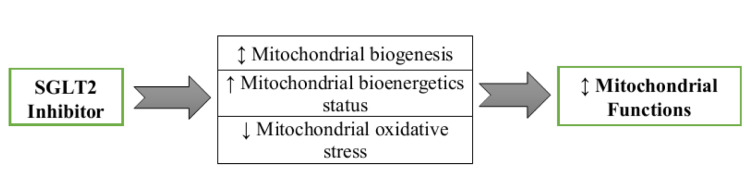
SGLT2 inhibitors modulate mitochondrial function through at least three pathways

SGLT2 inhibitors are a class of potent antidiabetic agents that exert extraglycemic effects through altering different physiologic systems such as mitochondria organelles. Emerging evidence has established that mitochondrial dysfunction is a major feature of a wide range of disorders and tissue dysfunctions. In this current study, we surveyed the literature for possible mitochondrial benefits of SGLT2 inhibitors and found that they can modulate mitochondrial functions through at least three pathways: by altering mitochondrial biogenesis and morphology, by control of mitochondrial ROS generation and by regulating mitochondrial ATP production (Figure 4(Fig. 4)). However, other potential pathways have been suggested that require further investigation. Conversely, we also highlight some adverse reports indicating disadvantageous effects of SGLT2 inhibitors on mitochondria. Further studies are needed to address these issues.

## Declaration

### Declarations

Ethics approval and consent to participate: not applicable to a review article.

### Consent for publication

All authors gave their consent for publication.

### Availability of data and materials

Not applicable as no novel data were generated for this review article.

### Competing interests

No authors have any conflict of interest or competing interests to declare.

### Funding

No funding was received to perform this study.

### Author contributions

HY and MM researched the data and wrote the first draft of the manuscript. AEB and TJ edited the manuscript. AS was responsible for conceptualization and supervision. All authors reviewed and approved the final version of the manuscript. Amirhossein Sahebkar is the guarantor of this work. 

### Acknowledgments

None.

## Figures and Tables

**Table 1:**
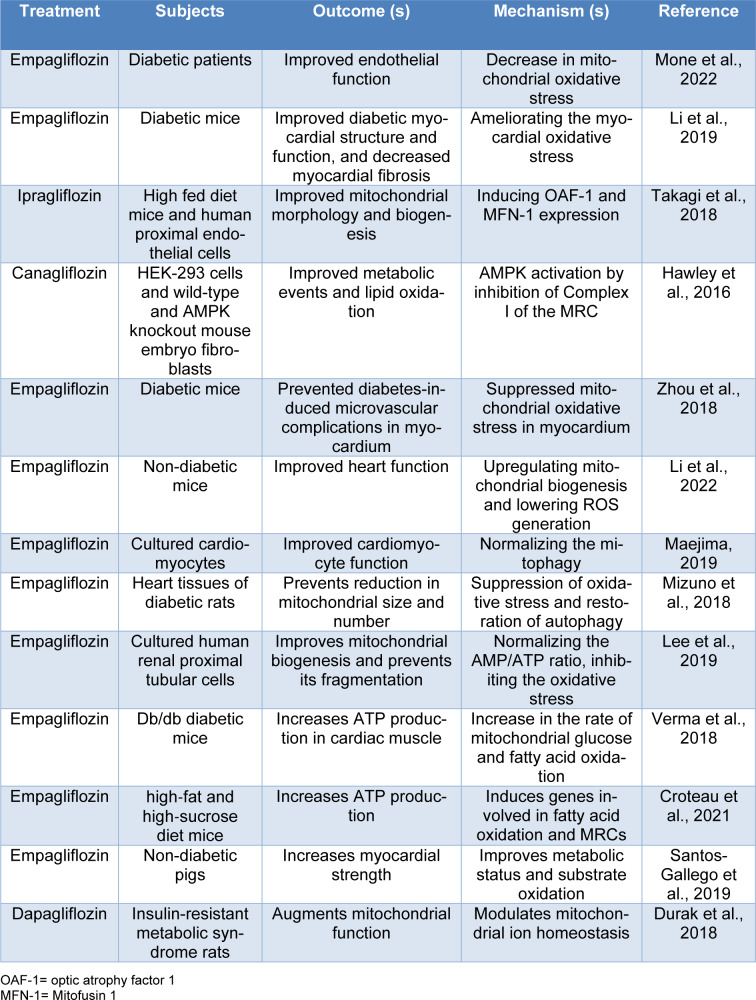
Summary of the main *in vitro *and* in vivo* evidence concerning the effects of SGLT2 inhibitors on mitochondrial function
